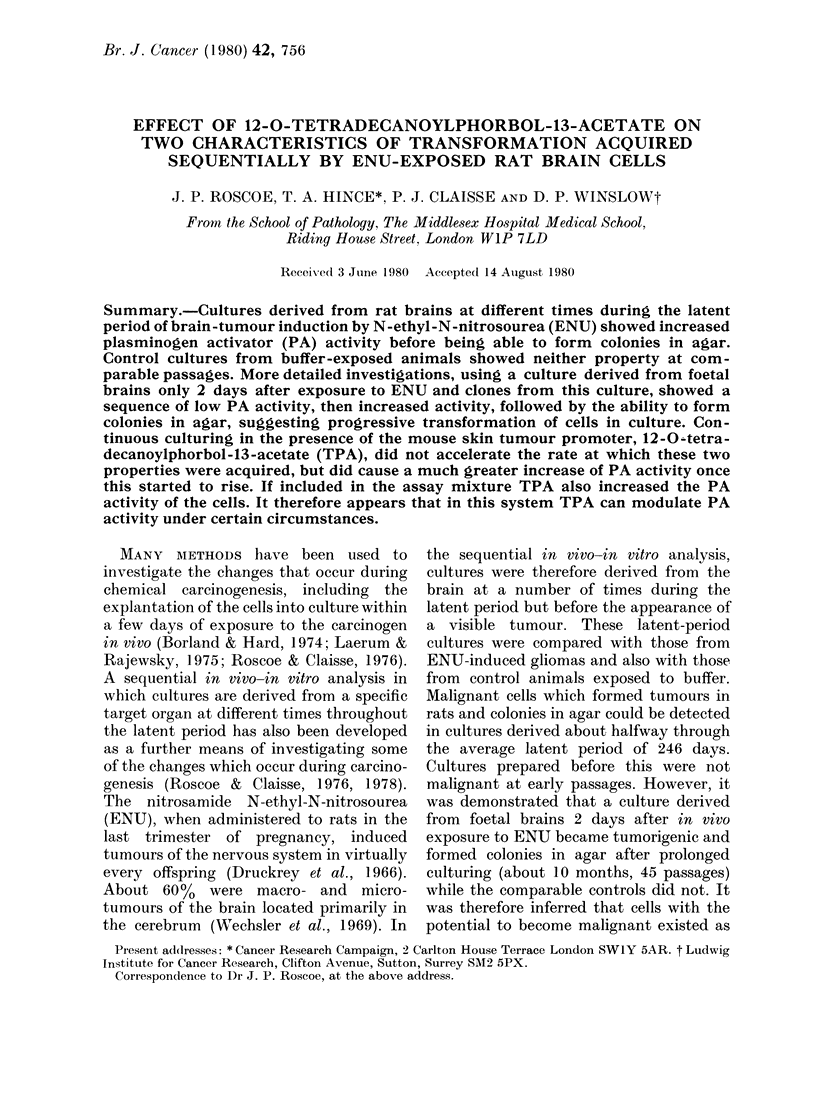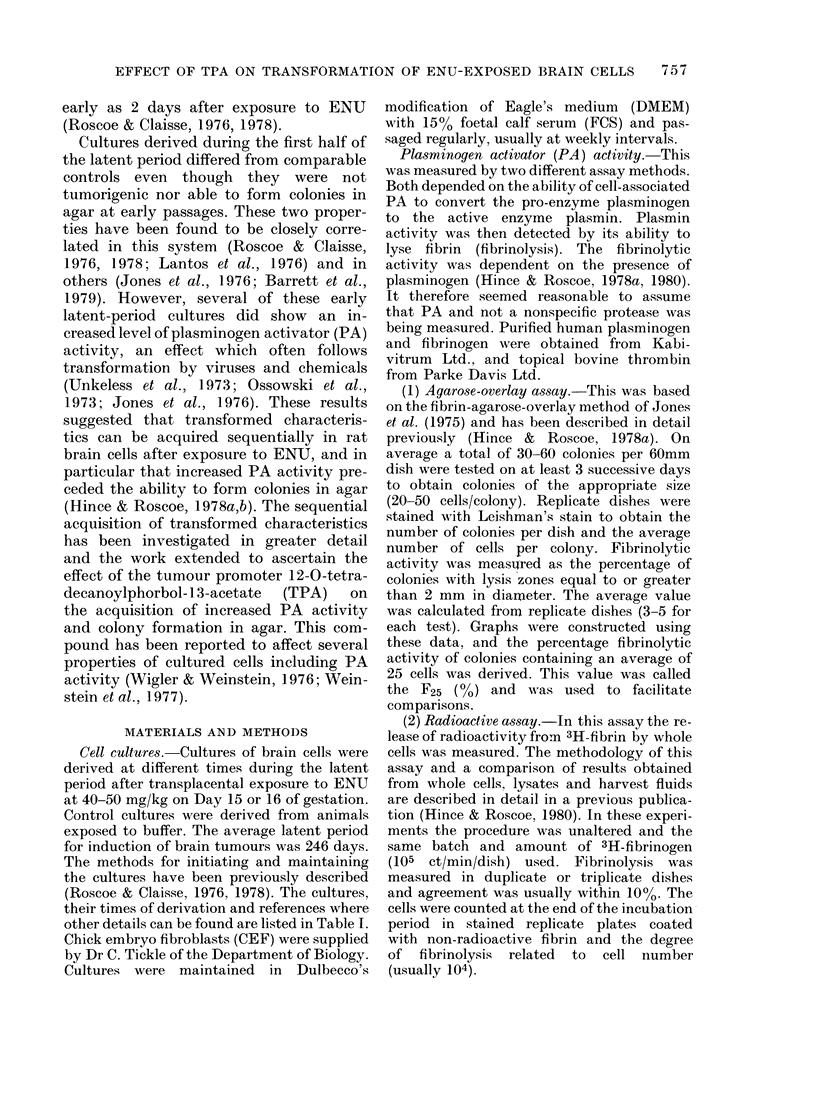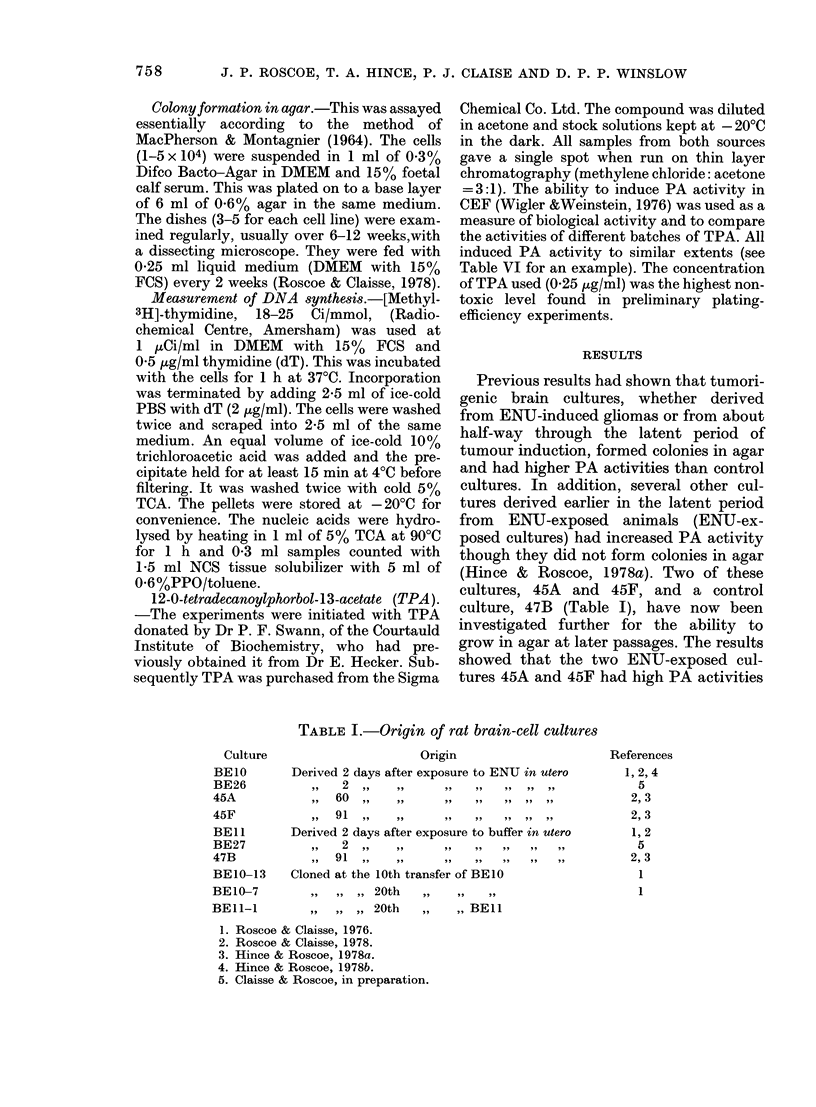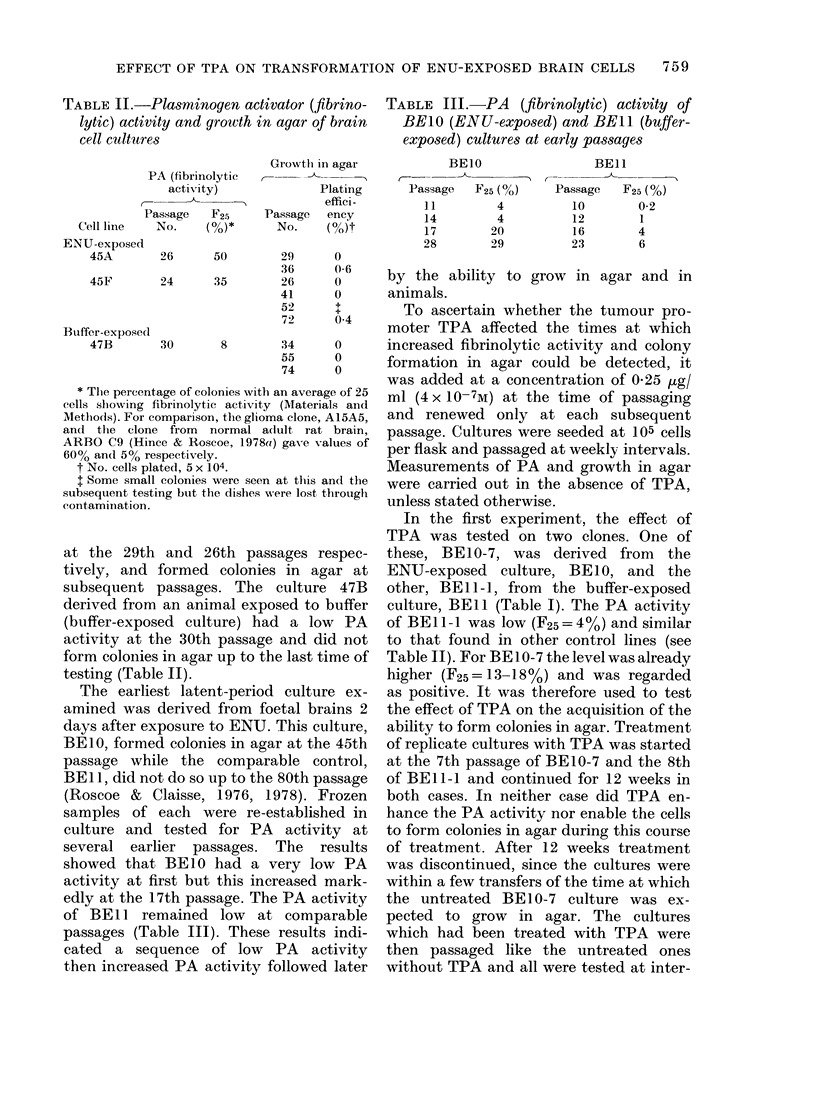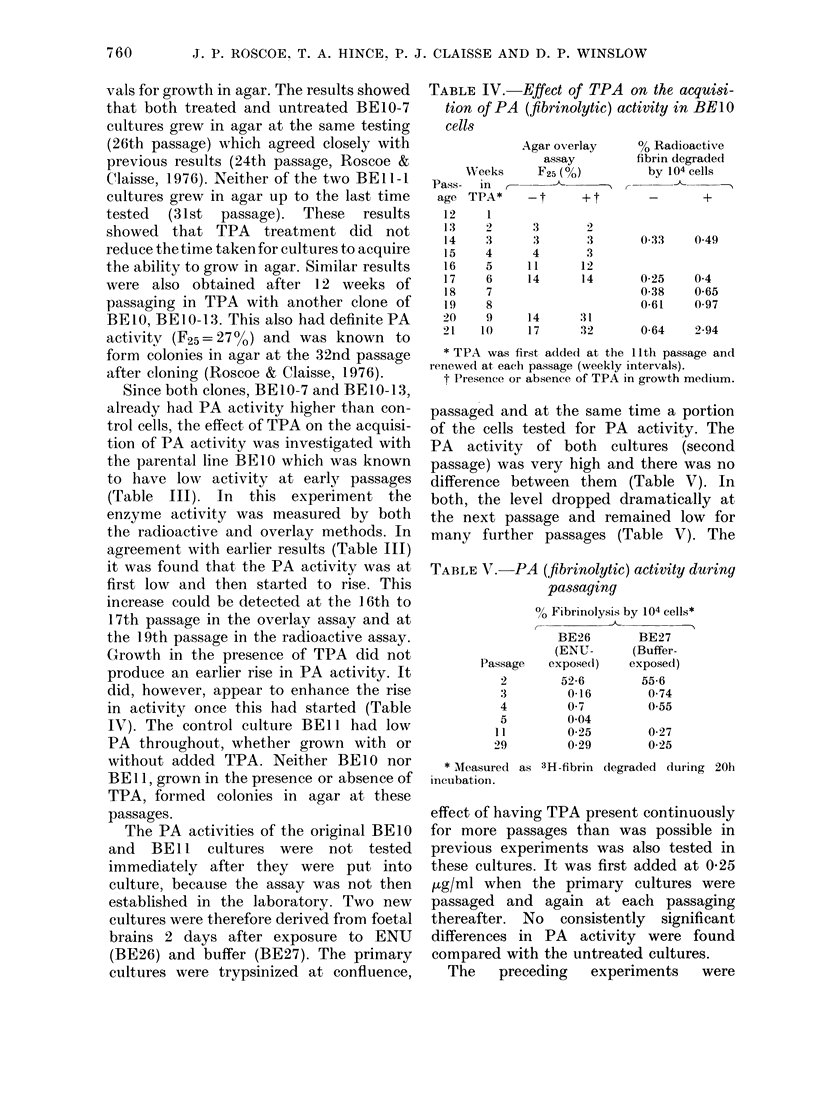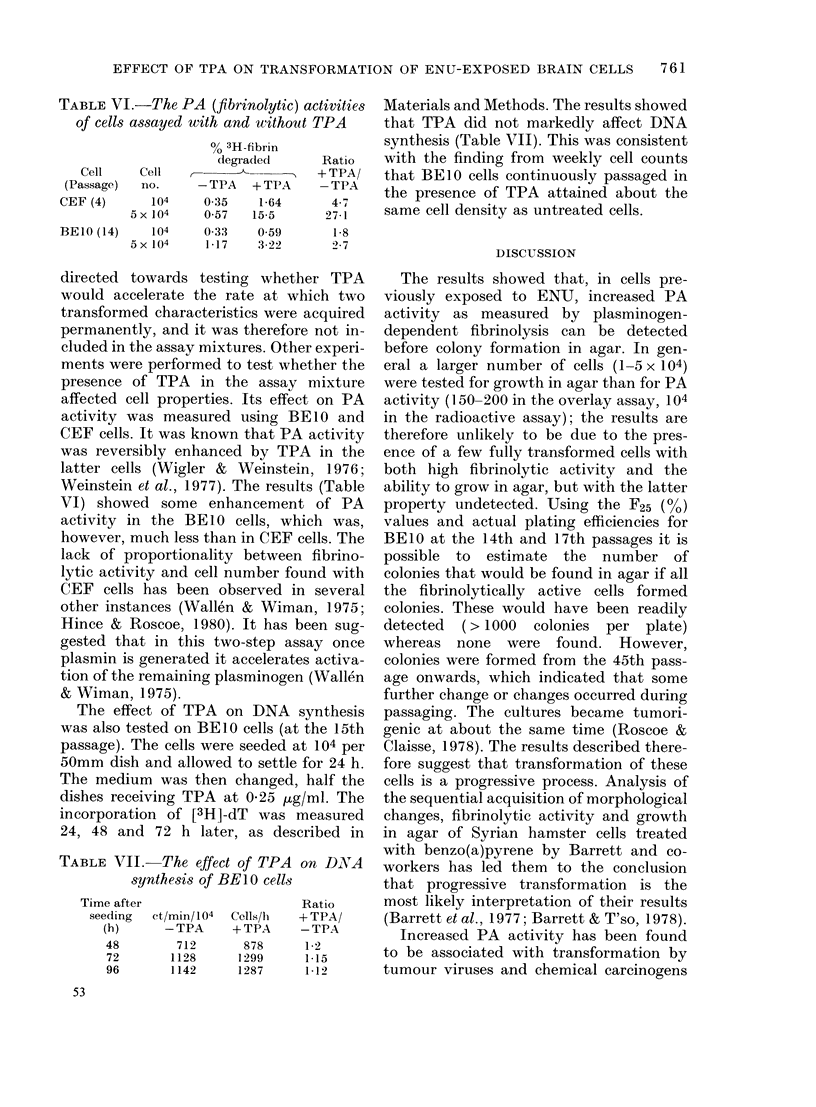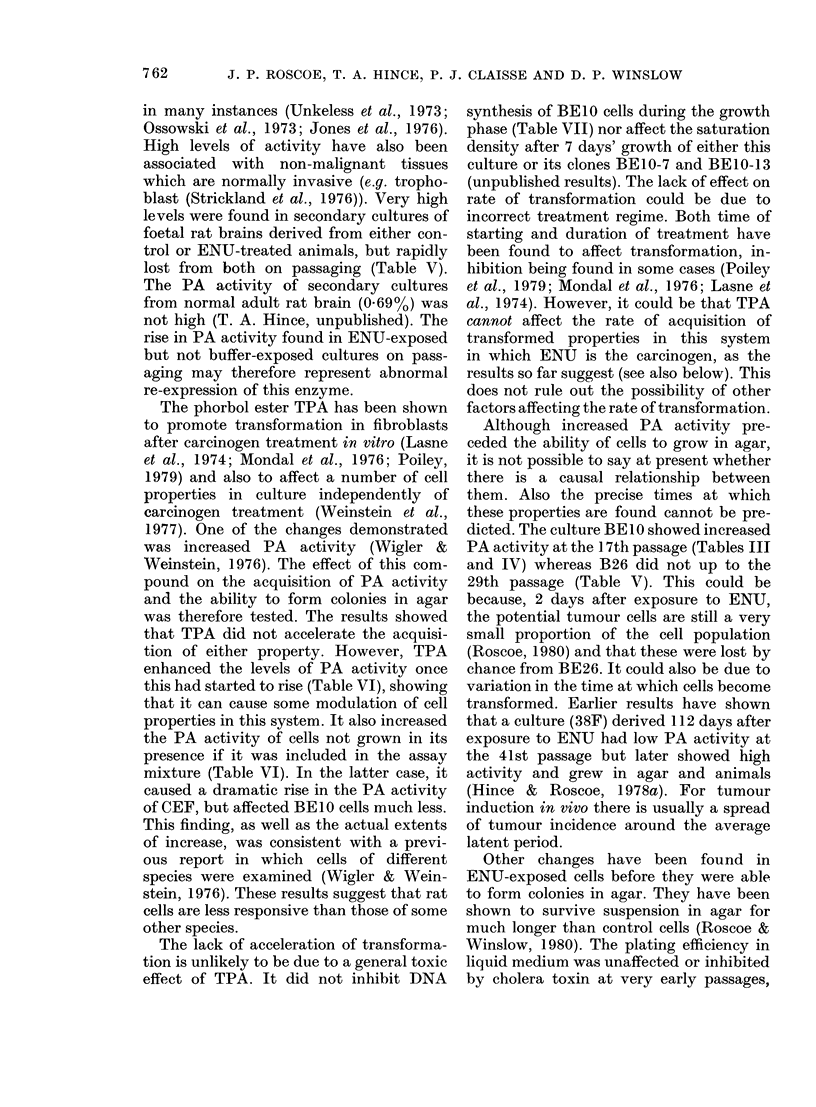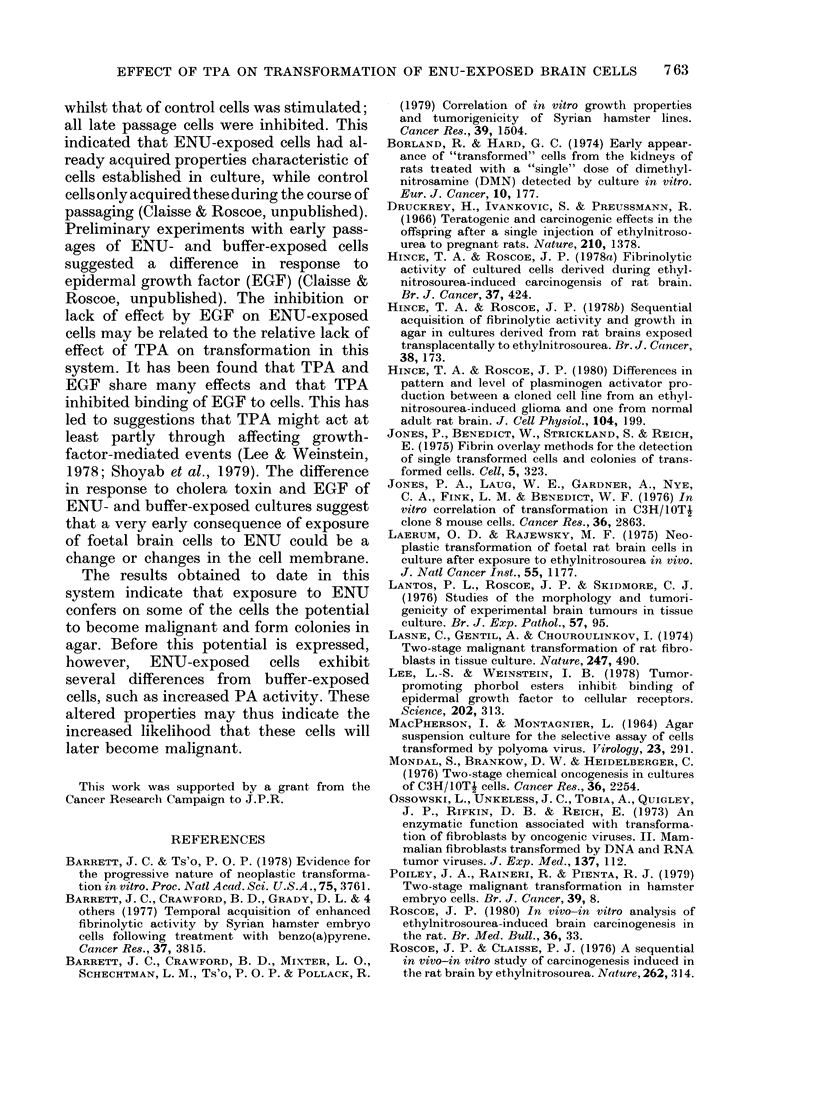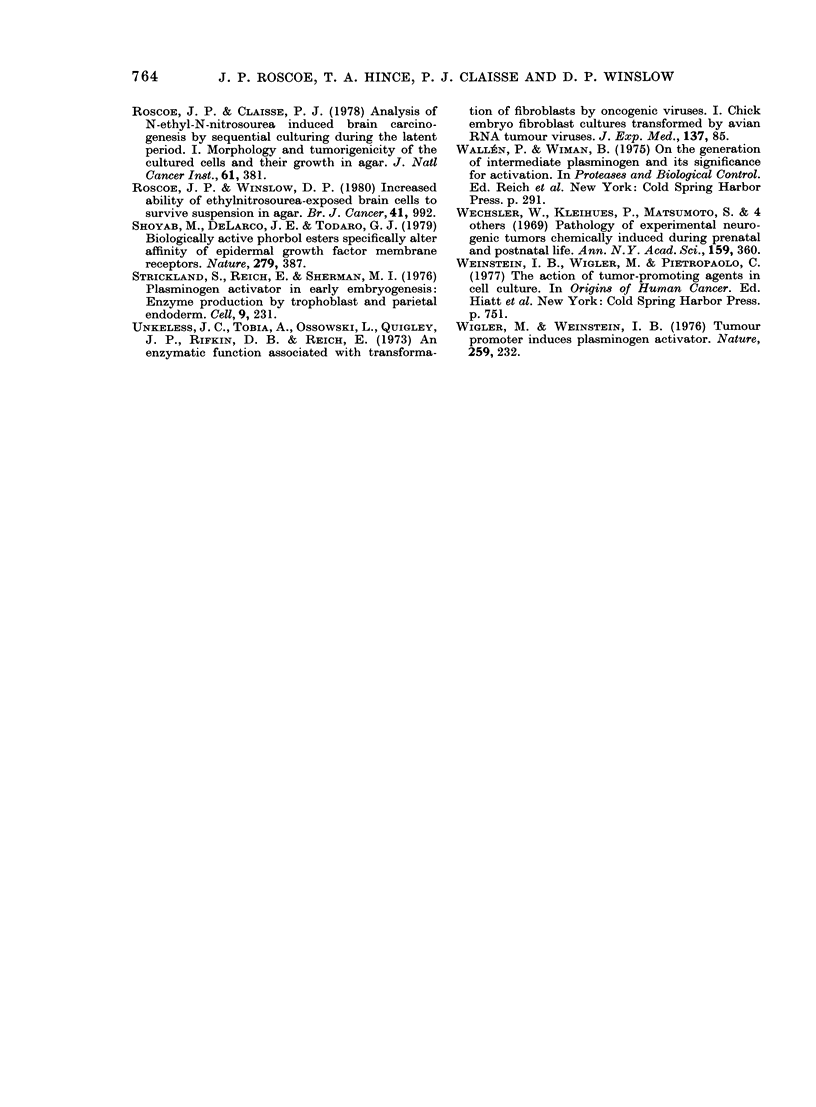# Effect of 12-O-tetradecanoylphorbol-13-acetate on two charateristics of transformation acquired sequentially by ENU-exposed rat brain cells.

**DOI:** 10.1038/bjc.1980.309

**Published:** 1980-11

**Authors:** J. P. Roscoe, T. A. Hince, P. J. Claisse, D. P. Winslow

## Abstract

Cultures derived from rat brains at different times during the latent period of brain-tumour induction by N-ethyl-N-nitrosourea (ENU) showed increased plasminogen activator (PA) activity before being able to form colonies in agar. Control cultures from buffer-exposed animals showed neither property at comparable passages. More detailed investigations, using a culture derived from foetal brains only 2 days after exposure to ENU and clones from this culture, showed a sequence of low PA activity, then increased activity, followed by the ability to form colonies in agar, suggesting progressive transformation of cells in culture. Continuous culturing in the presence of the mouse skin tumour promoter, 12-O-tetradecanoylphorbol-13-acetate (TPA), did not accelerate the rate at which these two properties were acquired, but did cause a much greater increase of PA activity once this started to rise. If included in the assay mixture TPA also increased the PA activity of the cells. It therefore appears that in this system TPA can modulate PA activity under certain circumstances.


Br. J. Cancer (1980) **42**, 756

### EFFECT OF 12-O-TETRADECANOYLPHORBOL-13-ACETATE ON TWO CHARACTERISTICS OF TRANSFORMATION ACQUIRED SEQUENTIALLY BY ENU-EXPOSED RAT BRAIN CELLS

J. P. ROSCOE, T. A. HINCE*, P. J. CLAISSE AND D. P. WINSLOW†

*From the School of Pathology, The Middlesex Hospital Medical School, Riding House Street, London W1P 7LD*

Received 3 June 1980 Accepted 14 August 1980

**Summary.—Cultures derived from rat brains at different times during the latent period of brain-tumour induction by N-ethyl-N-nitrosourea (ENU) showed increased plasminogen activator (PA) activity before being able to form colonies in agar. Control cultures from buffer-exposed animals showed neither property at comparable passages. More detailed investigations, using a culture derived from foetal brains only 2 days after exposure to ENU and clones from this culture, showed a sequence of low PA activity, then increased activity, followed by the ability to form colonies in agar, suggesting progressive transformation of cells in culture. Continuous culturing in the presence of the mouse skin tumour promoter, 12-O-tetradecanoylphorbol-13-acetate (TPA), did not accelerate the rate at which these two properties were acquired, but did cause a much greater increase of PA activity once this started to rise. If included in the assay mixture TPA also increased the PA activity of the cells. It therefore appears that in this system TPA can modulate PA activity under certain circumstances.**

MANY METHODS have been used to investigate the changes that occur during chemical carcinogenesis, including the explantation of the cells into culture within a few days of exposure to the carcinogen *in vivo* (Borland & Hard, 1974; Laerum & Rajewsky, 1975; Roscoe & Claisse, 1976). A sequential *in vivo–in vitro* analysis in which cultures are derived from a specific target organ at different times throughout the latent period has also been developed as a further means of investigating some of the changes which occur during carcinogenesis (Roscoe & Claisse, 1976, 1978). The nitrosamide N-ethyl-N-nitrosourea (ENU), when administered to rats in the last trimester of pregnancy, induced tumours of the nervous system in virtually every offspring (Druckrey *et al.*, 1966). About 60% were macro- and micro-tumours of the brain located primarily in the cerebrum (Wechsler *et al.*, 1969). In the sequential *in vivo–in vitro* analysis, cultures were therefore derived from the brain at a number of times during the latent period but before the appearance of a visible tumour. These latent-period cultures were compared with those from ENU-induced gliomas and also with those from control animals exposed to buffer. Malignant cells which formed tumours in rats and colonies in agar could be detected in cultures derived about halfway through the average latent period of 246 days. Cultures prepared before this were not malignant at early passages. However, it was demonstrated that a culture derived from foetal brains 2 days after *in vivo* exposure to ENU became tumorigenic and formed colonies in agar after prolonged culturing (about 10 months, 45 passages) while the comparable controls did not. It was therefore inferred that cells with the potential to become malignant existed as

Present addresses: *Cancer Research Campaign, 2 Carlton House Terrace London SW1Y 5AR. † Ludwig Institute for Cancer Research, Clifton Avenue, Sutton, Surrey SM2 5PX.
Correspondence to Dr J. P. Roscoe, at the above address.

early as 2 days after exposure to ENU (Roscoe & Claisse, 1976, 1978).

Cultures derived during the first half of the latent period differed from comparable controls even though they were not tumorigenic nor able to form colonies in agar at early passages. These two properties have been found to be closely correlated in this system (Roscoe & Claisse, 1976, 1978; Lantos *et al.*, 1976) and in others (Jones *et al.*, 1976; Barrett *et al.*, 1979). However, several of these early latent-period cultures did show an increased level of plasminogen activator (PA) activity, an effect which often follows transformation by viruses and chemicals (Unkeless *et al.*, 1973; Ossowski *et al.*, 1973; Jones *et al.*, 1976). These results suggested that transformed characteristics can be acquired sequentially in rat brain cells after exposure to ENU, and in particular that increased PA activity preceded the ability to form colonies in agar (Hince & Roscoe, 1978*a*,*b*). The sequential acquisition of transformed characteristics has been investigated in greater detail and the work extended to ascertain the effect of the tumour promoter 12-O-tetradecanoylphorbol-13-acetate (TPA) on the acquisition of increased PA activity and colony formation in agar. This compound has been reported to affect several properties of cultured cells including PA activity (Wigler & Weinstein, 1976; Weinstein *et al.*, 1977).

### MATERIALS AND METHODS

*Cell cultures.*—Cultures of brain cells were derived at different times during the latent period after transplacental exposure to ENU at 40–50 mg/kg on Day 15 or 16 of gestation. Control cultures were derived from animals exposed to buffer. The average latent period for induction of brain tumours was 246 days. The methods for initiating and maintaining the cultures have been previously described (Roscoe & Claisse, 1976, 1978). The cultures, their times of derivation and references where other details can be found are listed in Table I. Chick embryo fibroblasts (CEF) were supplied by Dr C. Tickle of the Department of Biology. Cultures were maintained in Dulbecco's modification of Eagle's medium (DMEM) with 15% foetal calf serum (FCS) and passaged regularly, usually at weekly intervals.

*Plasminogen activator (PA) activity.*—This was measured by two different assay methods. Both depended on the ability of cell-associated PA to convert the pro-enzyme plasminogen to the active enzyme plasmin. Plasmin activity was then detected by its ability to lyse fibrin (fibrinolysis). The fibrinolytic activity was dependent on the presence of plasminogen (Hince & Roscoe, 1978*a*, 1980). It therefore seemed reasonable to assume that PA and not a nonspecific protease was being measured. Purified human plasminogen and fibrinogen were obtained from Kabivitrum Ltd., and topical bovine thrombin from Parke Davis Ltd.

(1) *Agarose-overlay assay.*—This was based on the fibrin-agarose-overlay method of Jones *et al.* (1975) and has been described in detail previously (Hince & Roscoe, 1978*a*). On average a total of 30–60 colonies per 60mm dish were tested on at least 3 successive days to obtain colonies of the appropriate size (20–50 cells/colony). Replicate dishes were stained with Leishman's stain to obtain the number of colonies per dish and the average number of cells per colony. Fibrinolytic activity was measured as the percentage of colonies with lysis zones equal to or greater than 2 mm in diameter. The average value was calculated from replicate dishes (3–5 for each test). Graphs were constructed using these data, and the percentage fibrinolytic activity of colonies containing an average of 25 cells was derived. This value was called the $F_{25}$ (%) and was used to facilitate comparisons.

(2) *Radioactive assay.*—In this assay the release of radioactivity from $^3$H-fibrin by whole cells was measured. The methodology of this assay and a comparison of results obtained from whole cells, lysates and harvest fluids are described in detail in a previous publication (Hince & Roscoe, 1980). In these experiments the procedure was unaltered and the same batch and amount of $^3$H-fibrinogen ($10^5$ ct/min/dish) used. Fibrinolysis was measured in duplicate or triplicate dishes and agreement was usually within 10%. The cells were counted at the end of the incubation period in stained replicate plates coated with non-radioactive fibrin and the degree of fibrinolysis related to cell number (usually $10^4$).

*Colony formation in agar.*—This was assayed essentially according to the method of MacPherson & Montagnier (1964). The cells ($1–5 \times 10^4$) were suspended in 1 ml of 0·3% Difco Bacto-Agar in DMEM and 15% foetal calf serum. This was plated on to a base layer of 6 ml of 0·6% agar in the same medium. The dishes (3–5 for each cell line) were examined regularly, usually over 6–12 weeks, with a dissecting microscope. They were fed with 0·25 ml liquid medium (DMEM with 15% FCS) every 2 weeks (Roscoe & Claisse, 1978).

*Measurement of DNA synthesis.*—[Methyl-$^3$H]-thymidine, 18–25 Ci/mmol, (Radiochemical Centre, Amersham) was used at 1 $\mu$Ci/ml in DMEM with 15% FCS and 0·5 $\mu$g/ml thymidine (dT). This was incubated with the cells for 1 h at 37°C. Incorporation was terminated by adding 2·5 ml of ice-cold PBS with dT (2 $\mu$g/ml). The cells were washed twice and scraped into 2·5 ml of the same medium. An equal volume of ice-cold 10% trichloroacetic acid was added and the precipitate held for at least 15 min at 4°C before filtering. It was washed twice with cold 5% TCA. The pellets were stored at −20°C for convenience. The nucleic acids were hydrolysed by heating in 1 ml of 5% TCA at 90°C for 1 h and 0·3 ml samples counted with 1·5 ml NCS tissue solubilizer with 5 ml of 0·6%PPO/toluene.

12-0-*tetradecanoylphorbol*-13-*acetate* (*TPA*).—The experiments were initiated with TPA donated by Dr P. F. Swann, of the Courtauld Institute of Biochemistry, who had previously obtained it from Dr E. Hecker. Subsequently TPA was purchased from the Sigma Chemical Co. Ltd. The compound was diluted in acetone and stock solutions kept at −20°C in the dark. All samples from both sources gave a single spot when run on thin layer chromatography (methylene chloride: acetone = 3:1). The ability to induce PA activity in CEF (Wigler & Weinstein, 1976) was used as a measure of biological activity and to compare the activities of different batches of TPA. All induced PA activity to similar extents (see Table VI for an example). The concentration of TPA used (0·25 $\mu$g/ml) was the highest non-toxic level found in preliminary plating-efficiency experiments.

### RESULTS

Previous results had shown that tumorigenic brain cultures, whether derived from ENU-induced gliomas or from about half-way through the latent period of tumour induction, formed colonies in agar and had higher PA activities than control cultures. In addition, several other cultures derived earlier in the latent period from ENU-exposed animals (ENU-exposed cultures) had increased PA activity though they did not form colonies in agar (Hince & Roscoe, 1978*a*). Two of these cultures, 45A and 45F, and a control culture, 47B (Table I), have now been investigated further for the ability to grow in agar at later passages. The results showed that the two ENU-exposed cultures 45A and 45F had high PA activities

TABLE I.—*Origin of rat brain-cell cultures*

| Culture | Origin | References |
|---|---|---|
| BE10 | Derived 2 days after exposure to ENU *in utero* | 1, 2, 4 |
| BE26 | ,, 2 ,, ,, ,, ,, ,, ,, ,, | 5 |
| 45A | ,, 60 ,, ,, ,, ,, ,, ,, ,, | 2, 3 |
| 45F | ,, 91 ,, ,, ,, ,, ,, ,, ,, | 2, 3 |
| BE11 | Derived 2 days after exposure to buffer *in utero* | 1, 2 |
| BE27 | ,, 2 ,, ,, ,, ,, ,, ,, ,, | 5 |
| 47B | ,, 91 ,, ,, ,, ,, ,, ,, ,, | 2, 3 |
| BE10-13 | Cloned at the 10th transfer of BE10 | 1 |
| BE10-7 | ,, ,, ,, 20th ,, ,, ,, | 1 |
| BE11-1 | ,, ,, ,, 20th ,, ,, BE11 | |

1. Roscoe & Claisse, 1976.
2. Roscoe & Claisse, 1978.
3. Hince & Roscoe, 1978*a*.
4. Hince & Roscoe, 1978*b*.
5. Claisse & Roscoe, in preparation.

TABLE II.—*Plasminogen activator (fibrinolytic) activity and growth in agar of brain cell cultures*

| Cell line | PA (fibrinolytic activity) Passage No. | $F_{25}$ (%)* | Growth in agar Passage No. | Plating efficiency (%)† |
|---|---|---|---|---|
| ENU-exposed | | | | |
| 45A | 26 | 50 | 29 | 0 |
| | | | 36 | 0·6 |
| 45F | 24 | 35 | 26 | 0 |
| | | | 41 | 0 |
| | | | 52 | ‡ |
| | | | 72 | 0·4 |
| Buffer-exposed | | | | |
| 47B | 30 | 8 | 34 | 0 |
| | | | 55 | 0 |
| | | | 74 | 0 |

* The percentage of colonies with an average of 25 cells showing fibrinolytic activity (Materials and Methods). For comparison, the glioma clone, A15A5, and the clone from normal adult rat brain, ARBO C9 (Hince & Roscoe, 1978*a*) gave values of 60% and 5% respectively.

† No. cells plated, $5 \times 10^4$.

‡ Some small colonies were seen at this and the subsequent testing but the dishes were lost through contamination.

at the 29th and 26th passages respectively, and formed colonies in agar at subsequent passages. The culture 47B derived from an animal exposed to buffer (buffer-exposed culture) had a low PA activity at the 30th passage and did not form colonies in agar up to the last time of testing (Table II).

The earliest latent-period culture examined was derived from foetal brains 2 days after exposure to ENU. This culture, BE10, formed colonies in agar at the 45th passage while the comparable control, BE11, did not do so up to the 80th passage (Roscoe & Claisse, 1976, 1978). Frozen samples of each were re-established in culture and tested for PA activity at several earlier passages. The results showed that BE10 had a very low PA activity at first but this increased markedly at the 17th passage. The PA activity of BE11 remained low at comparable passages (Table III). These results indicated a sequence of low PA activity then increased PA activity followed later

TABLE III.—*PA (fibrinolytic) activity of BE10 (ENU-exposed) and BE11 (buffer-exposed) cultures at early passages*

| BE10 Passage | BE10 $F_{25}$ (%) | BE11 Passage | BE11 $F_{25}$ (%) |
|---|---|---|---|
| 11 | 4 | 10 | 0·2 |
| 14 | 4 | 12 | 1 |
| 17 | 20 | 16 | 4 |
| 28 | 29 | 23 | 6 |

by the ability to grow in agar and in animals.

To ascertain whether the tumour promoter TPA affected the times at which increased fibrinolytic activity and colony formation in agar could be detected, it was added at a concentration of 0·25 $\mu$g/ml ($4 \times 10^{-7}$M) at the time of passaging and renewed only at each subsequent passage. Cultures were seeded at $10^5$ cells per flask and passaged at weekly intervals. Measurements of PA and growth in agar were carried out in the absence of TPA, unless stated otherwise.

In the first experiment, the effect of TPA was tested on two clones. One of these, BE10-7, was derived from the ENU-exposed culture, BE10, and the other, BE11-1, from the buffer-exposed culture, BE11 (Table I). The PA activity of BE11-1 was low ($F_{25} = 4\%$) and similar to that found in other control lines (see Table II). For BE10-7 the level was already higher ($F_{25} = 13$–$18\%$) and was regarded as positive. It was therefore used to test the effect of TPA on the acquisition of the ability to form colonies in agar. Treatment of replicate cultures with TPA was started at the 7th passage of BE10-7 and the 8th of BE11-1 and continued for 12 weeks in both cases. In neither case did TPA enhance the PA activity nor enable the cells to form colonies in agar during this course of treatment. After 12 weeks treatment was discontinued, since the cultures were within a few transfers of the time at which the untreated BE10-7 culture was expected to grow in agar. The cultures which had been treated with TPA were then passaged like the untreated ones without TPA and all were tested at intervals for growth in agar. The results showed that both treated and untreated BE10-7 cultures grew in agar at the same testing (26th passage) which agreed closely with previous results (24th passage, Roscoe & Claisse, 1976). Neither of the two BE11-1 cultures grew in agar up to the last time tested (31st passage). These results showed that TPA treatment did not reduce the time taken for cultures to acquire the ability to grow in agar. Similar results were also obtained after 12 weeks of passaging in TPA with another clone of BE10, BE10-13. This also had definite PA activity ($F_{25} = 27\%$) and was known to form colonies in agar at the 32nd passage after cloning (Roscoe & Claisse, 1976).

Since both clones, BE10-7 and BE10-13, already had PA activity higher than control cells, the effect of TPA on the acquisition of PA activity was investigated with the parental line BE10 which was known to have low activity at early passages (Table III). In this experiment the enzyme activity was measured by both the radioactive and overlay methods. In agreement with earlier results (Table III) it was found that the PA activity was at first low and then started to rise. This increase could be detected at the 16th to 17th passage in the overlay assay and at the 19th passage in the radioactive assay. Growth in the presence of TPA did not produce an earlier rise in PA activity. It did, however, appear to enhance the rise in activity once this had started (Table IV). The control culture BE11 had low PA throughout, whether grown with or without added TPA. Neither BE10 nor BE11, grown in the presence or absence of TPA, formed colonies in agar at these passages.

The PA activities of the original BE10 and BE11 cultures were not tested immediately after they were put into culture, because the assay was not then established in the laboratory. Two new cultures were therefore derived from foetal brains 2 days after exposure to ENU (BE26) and buffer (BE27). The primary cultures were trypsinized at confluence,

TABLE IV.—*Effect of TPA on the acquisition of PA (fibrinolytic) activity in BE*10 *cells*

| Passage | Weeks in TPA* | Agar overlay assay $F_{25}$ (%) −† | +† | % Radioactive fibrin degraded by $10^4$ cells − | + |
|---|---|---|---|---|---|
| 12 | 1 | | | | |
| 13 | 2 | 3 | 2 | | |
| 14 | 3 | 3 | 3 | 0·33 | 0·49 |
| 15 | 4 | 4 | 3 | | |
| 16 | 5 | 11 | 12 | | |
| 17 | 6 | 14 | 14 | 0·25 | 0·4 |
| 18 | 7 | | | 0·38 | 0·65 |
| 19 | 8 | | | 0·61 | 0·97 |
| 20 | 9 | 14 | 31 | | |
| 21 | 10 | 17 | 32 | 0·64 | 2·94 |

* TPA was first added at the 11th passage and renewed at each passage (weekly intervals).
† Presence or absence of TPA in growth medium.

passaged and at the same time a portion of the cells tested for PA activity. The PA activity of both cultures (second passage) was very high and there was no difference between them (Table V). In both, the level dropped dramatically at the next passage and remained low for many further passages (Table V). The effect of having TPA present continuously for more passages than was possible in previous experiments was also tested in these cultures. It was first added at 0·25 μg/ml when the primary cultures were passaged and again at each passaging thereafter. No consistently significant differences in PA activity were found compared with the untreated cultures.

TABLE V.—*PA (fibrinolytic) activity during passaging*

| Passage | % Fibrinolysis by $10^4$ cells* BE26 (ENU-exposed) | BE27 (Buffer-exposed) |
|---|---|---|
| 2 | 52·6 | 55·6 |
| 3 | 0·16 | 0·74 |
| 4 | 0·7 | 0·55 |
| 5 | 0·04 | |
| 11 | 0·25 | 0·27 |
| 29 | 0·29 | 0·25 |

* Measured as $^3$H-fibrin degraded during 20h incubation.

The preceding experiments were

TABLE VI.—*The PA (fibrinolytic) activities of cells assayed with and without TPA*

| Cell (Passage) | Cell no. | % $^3$H-fibrin degraded −TPA | +TPA | Ratio +TPA/ −TPA |
|---|---|---|---|---|
| CEF (4) | $10^4$ | 0·35 | 1·64 | 4·7 |
| | $5 \times 10^4$ | 0·57 | 15·5 | 27·1 |
| BE10 (14) | $10^4$ | 0·33 | 0·59 | 1·8 |
| | $5 \times 10^4$ | 1·17 | 3·22 | 2·7 |

directed towards testing whether TPA would accelerate the rate at which two transformed characteristics were acquired permanently, and it was therefore not included in the assay mixtures. Other experiments were performed to test whether the presence of TPA in the assay mixture affected cell properties. Its effect on PA activity was measured using BE10 and CEF cells. It was known that PA activity was reversibly enhanced by TPA in the latter cells (Wigler & Weinstein, 1976; Weinstein *et al.*, 1977). The results (Table VI) showed some enhancement of PA activity in the BE10 cells, which was, however, much less than in CEF cells. The lack of proportionality between fibrinolytic activity and cell number found with CEF cells has been observed in several other instances (Wallén & Wiman, 1975; Hince & Roscoe, 1980). It has been suggested that in this two-step assay once plasmin is generated it accelerates activation of the remaining plasminogen (Wallén & Wiman, 1975).

The effect of TPA on DNA synthesis was also tested on BE10 cells (at the 15th passage). The cells were seeded at $10^4$ per 50mm dish and allowed to settle for 24 h. The medium was then changed, half the dishes receiving TPA at 0·25 μg/ml. The incorporation of [$^3$H]-dT was measured 24, 48 and 72 h later, as described in Materials and Methods. The results showed that TPA did not markedly affect DNA synthesis (Table VII). This was consistent with the finding from weekly cell counts that BE10 cells continuously passaged in the presence of TPA attained about the same cell density as untreated cells.

TABLE VII.—*The effect of TPA on DNA synthesis of BE10 cells*

| Time after seeding (h) | ct/min/$10^4$ −TPA | Cells/h +TPA | Ratio +TPA/ −TPA |
|---|---|---|---|
| 48 | 712 | 878 | 1·2 |
| 72 | 1128 | 1299 | 1·15 |
| 96 | 1142 | 1287 | 1·12 |

### DISCUSSION

The results showed that, in cells previously exposed to ENU, increased PA activity as measured by plasminogen-dependent fibrinolysis can be detected before colony formation in agar. In general a larger number of cells ($1–5 \times 10^4$) were tested for growth in agar than for PA activity (150–200 in the overlay assay, $10^4$ in the radioactive assay); the results are therefore unlikely to be due to the presence of a few fully transformed cells with both high fibrinolytic activity and the ability to grow in agar, but with the latter property undetected. Using the $F_{25}$ (%) values and actual plating efficiencies for BE10 at the 14th and 17th passages it is possible to estimate the number of colonies that would be found in agar if all the fibrinolytically active cells formed colonies. These would have been readily detected (>1000 colonies per plate) whereas none were found. However, colonies were formed from the 45th passage onwards, which indicated that some further change or changes occurred during passaging. The cultures became tumorigenic at about the same time (Roscoe & Claisse, 1978). The results described therefore suggest that transformation of these cells is a progressive process. Analysis of the sequential acquisition of morphological changes, fibrinolytic activity and growth in agar of Syrian hamster cells treated with benzo(a)pyrene by Barrett and co-workers has led them to the conclusion that progressive transformation is the most likely interpretation of their results (Barrett *et al.*, 1977; Barrett & T'so, 1978).

Increased PA activity has been found to be associated with transformation by tumour viruses and chemical carcinogens in many instances (Unkeless *et al.*, 1973; Ossowski *et al.*, 1973; Jones *et al.*, 1976). High levels of activity have also been associated with non-malignant tissues which are normally invasive (*e.g.* trophoblast (Strickland *et al.*, 1976)). Very high levels were found in secondary cultures of foetal rat brains derived from either control or ENU-treated animals, but rapidly lost from both on passaging (Table V). The PA activity of secondary cultures from normal adult rat brain (0·69%) was not high (T. A. Hince, unpublished). The rise in PA activity found in ENU-exposed but not buffer-exposed cultures on passaging may therefore represent abnormal re-expression of this enzyme.

The phorbol ester TPA has been shown to promote transformation in fibroblasts after carcinogen treatment *in vitro* (Lasne *et al.*, 1974; Mondal *et al.*, 1976; Poiley, 1979) and also to affect a number of cell properties in culture independently of carcinogen treatment (Weinstein *et al.*, 1977). One of the changes demonstrated was increased PA activity (Wigler & Weinstein, 1976). The effect of this compound on the acquisition of PA activity and the ability to form colonies in agar was therefore tested. The results showed that TPA did not accelerate the acquisition of either property. However, TPA enhanced the levels of PA activity once this had started to rise (Table VI), showing that it can cause some modulation of cell properties in this system. It also increased the PA activity of cells not grown in its presence if it was included in the assay mixture (Table VI). In the latter case, it caused a dramatic rise in the PA activity of CEF, but affected BE10 cells much less. This finding, as well as the actual extents of increase, was consistent with a previous report in which cells of different species were examined (Wigler & Weinstein, 1976). These results suggest that rat cells are less responsive than those of some other species.

The lack of acceleration of transformation is unlikely to be due to a general toxic effect of TPA. It did not inhibit DNA synthesis of BE10 cells during the growth phase (Table VII) nor affect the saturation density after 7 days' growth of either this culture or its clones BE10-7 and BE10-13 (unpublished results). The lack of effect on rate of transformation could be due to incorrect treatment regime. Both time of starting and duration of treatment have been found to affect transformation, inhibition being found in some cases (Poiley *et al.*, 1979; Mondal *et al.*, 1976; Lasne *et al.*, 1974). However, it could be that TPA *cannot* affect the rate of acquisition of transformed properties in this system in which ENU is the carcinogen, as the results so far suggest (see also below). This does not rule out the possibility of other factors affecting the rate of transformation.

Although increased PA activity preceded the ability of cells to grow in agar, it is not possible to say at present whether there is a causal relationship between them. Also the precise times at which these properties are found cannot be predicted. The culture BE10 showed increased PA activity at the 17th passage (Tables III and IV) whereas B26 did not up to the 29th passage (Table V). This could be because, 2 days after exposure to ENU, the potential tumour cells are still a very small proportion of the cell population (Roscoe, 1980) and that these were lost by chance from BE26. It could also be due to variation in the time at which cells become transformed. Earlier results have shown that a culture (38F) derived 112 days after exposure to ENU had low PA activity at the 41st passage but later showed high activity and grew in agar and animals (Hince & Roscoe, 1978*a*). For tumour induction *in vivo* there is usually a spread of tumour incidence around the average latent period.

Other changes have been found in ENU-exposed cells before they were able to form colonies in agar. They have been shown to survive suspension in agar for much longer than control cells (Roscoe & Winslow, 1980). The plating efficiency in liquid medium was unaffected or inhibited by cholera toxin at very early passages, whilst that of control cells was stimulated; all late passage cells were inhibited. This indicated that ENU-exposed cells had already acquired properties characteristic of cells established in culture, while control cells only acquired these during the course of passaging (Claisse & Roscoe, unpublished). Preliminary experiments with early passages of ENU- and buffer-exposed cells suggested a difference in response to epidermal growth factor (EGF) (Claisse & Roscoe, unpublished). The inhibition or lack of effect by EGF on ENU-exposed cells may be related to the relative lack of effect of TPA on transformation in this system. It has been found that TPA and EGF share many effects and that TPA inhibited binding of EGF to cells. This has led to suggestions that TPA might act at least partly through affecting growth-factor-mediated events (Lee & Weinstein, 1978; Shoyab *et al.*, 1979). The difference in response to cholera toxin and EGF of ENU- and buffer-exposed cultures suggest that a very early consequence of exposure of foetal brain cells to ENU could be a change or changes in the cell membrane.

The results obtained to date in this system indicate that exposure to ENU confers on some of the cells the potential to become malignant and form colonies in agar. Before this potential is expressed, however, ENU-exposed cells exhibit several differences from buffer-exposed cells, such as increased PA activity. These altered properties may thus indicate the increased likelihood that these cells will later become malignant.

This work was supported by a grant from the Cancer Research Campaign to J.P.R.

REFERENCES

BARRETT, J. C. & TS'O, P. O. P. (1978) Evidence for the progressive nature of neoplastic transformation *in vitro*. *Proc. Natl Acad. Sci. U.S.A.*, **75**, 3761.

BARRETT, J. C., CRAWFORD, B. D., GRADY, D. L. & 4 others (1977) Temporal acquisition of enhanced fibrinolytic activity by Syrian hamster embryo cells following treatment with benzo(a)pyrene. *Cancer Res.*, **37**, 3815.

BARRETT, J. C., CRAWFORD, B. D., MIXTER, L. O., SCHECHTMAN, L. M., TS'O, P. O. P. & POLLACK, R. (1979) Correlation of *in vitro* growth properties and tumorigenicity of Syrian hamster lines. *Cancer Res.*, **39**, 1504.

BORLAND, R. & HARD, G. C. (1974) Early appearance of "transformed" cells from the kidneys of rats treated with a "single" dose of dimethylnitrosamine (DMN) detected by culture *in vitro*. *Eur. J. Cancer*, **10**, 177.

DRUCKREY, H., IVANKOVIC, S. & PREUSSMANN, R. (1966) Teratogenic and carcinogenic effects in the offspring after a single injection of ethylnitrosourea to pregnant rats. *Nature*, **210**, 1378.

HINCE, T. A. & ROSCOE, J. P. (1978*a*) Fibrinolytic activity of cultured cells derived during ethylnitrosourea-induced carcinogensis of rat brain. *Br. J. Cancer*, **37**, 424.

HINCE, T. A. & ROSCOE, J. P. (1978*b*) Sequential acquisition of fibrinolytic activity and growth in agar in cultures derived from rat brains exposed transplacentally to ethylnitrosourea. *Br. J. Cancer*, **38**, 173.

HINCE, T. A. & ROSCOE, J. P. (1980) Differences in pattern and level of plasminogen activator production between a cloned cell line from an ethylnitrosourea-induced glioma and one from normal adult rat brain. *J. Cell Physiol.*, **104**, 199.

JONES, P., BENEDICT, W., STRICKLAND, S. & REICH, E. (1975) Fibrin overlay methods for the detection of single transformed cells and colonies of transformed cells. *Cell*, **5**, 323.

JONES, P. A., LAUG, W. E., GARDNER, A., NYE, C. A., FINK, L. M. & BENEDICT, W. F. (1976) *In vitro* correlation of transformation in C3H/10T½ clone 8 mouse cells. *Cancer Res.*, **36**, 2863.

LAERUM, O. D. & RAJEWSKY, M. F. (1975) Neoplastic transformation of foetal rat brain cells in culture after exposure to ethylnitrosourea *in vivo*. *J. Natl Cancer Inst.*, **55**, 1177.

LANTOS, P. L., ROSCOE, J. P. & SKIDMORE, C. J. (1976) Studies of the morphology and tumorigenicity of experimental brain tumours in tissue culture. *Br. J. Exp. Pathol.*, **57**, 95.

LASNE, C., GENTIL, A. & CHOUROULINKOV, I. (1974) Two-stage malignant transformation of rat fibroblasts in tissue culture. *Nature*, **247**, 490.

LEE, L.-S. & WEINSTEIN, I. B. (1978) Tumor-promoting phorbol esters inhibit binding of epidermal growth factor to cellular receptors. *Science*, **202**, 313.

MACPHERSON, I. & MONTAGNIER, L. (1964) Agar suspension culture for the selective assay of cells transformed by polyoma virus. *Virology*, **23**, 291.

MONDAL, S., BRANKOW, D. W. & HEIDELBERGER, C. (1976) Two-stage chemical oncogenesis in cultures of C3H/10T½ cells. *Cancer Res.*, **36**, 2254.

OSSOWSKI, L., UNKELESS, J. C., TOBIA, A., QUIGLEY, J. P., RIFKIN, D. B. & REICH, E. (1973) An enzymatic function associated with transformation of fibroblasts by oncogenic viruses. II. Mammalian fibroblasts transformed by DNA and RNA tumor viruses. *J. Exp. Med.*, **137**, 112.

POILEY, J. A., RAINERI, R. & PIENTA, R. J. (1979) Two-stage malignant transformation in hamster embryo cells. *Br. J. Cancer*, **39**, 8.

ROSCOE, J. P. (1980) *In vivo–in vitro* analysis of ethylnitrosourea-induced brain carcinogenesis in the rat. *Br. Med. Bull.*, **36**, 33.

ROSCOE, J. P. & CLAISSE, P. J. (1976) A sequential *in vivo–in vitro* study of carcinogenesis induced in the rat brain by ethylnitrosourea. *Nature*, **262**, 314.

ROSCOE, J. P. & CLAISSE, P. J. (1978) Analysis of N-ethyl-N-nitrosourea induced brain carcinogenesis by sequential culturing during the latent period. I. Morphology and tumorigenicity of the cultured cells and their growth in agar. *J. Natl Cancer Inst.*, **61**, 381.

ROSCOE, J. P. & WINSLOW, D. P. (1980) Increased ability of ethylnitrosourea-exposed brain cells to survive suspension in agar. *Br. J. Cancer*, **41**, 992.

SHOYAB, M., DELARCO, J. E. & TODARO, G. J. (1979) Biologically active phorbol esters specifically alter affinity of epidermal growth factor membrane receptors. *Nature*, **279**, 387.

STRICKLAND, S., REICH, E. & SHERMAN, M. I. (1976) Plasminogen activator in early embryogenesis: Enzyme production by trophoblast and parietal endoderm. *Cell*, **9**, 231.

UNKELESS, J. C., TOBIA, A., OSSOWSKI, L., QUIGLEY, J. P., RIFKIN, D. B. & REICH, E. (1973) An enzymatic function associated with transformation of fibroblasts by oncogenic viruses. I. Chick embryo fibroblast cultures transformed by avian RNA tumour viruses. *J. Exp. Med.*, **137**, 85.

WALLÉN, P. & WIMAN, B. (1975) On the generation of intermediate plasminogen and its significance for activation. In *Proteases and Biological Control.* Ed. Reich *et al.* New York: Cold Spring Harbor Press. p. 291.

WECHSLER, W., KLEIHUES, P., MATSUMOTO, S. & 4 others (1969) Pathology of experimental neurogenic tumors chemically induced during prenatal and postnatal life. *Ann. N.Y. Acad. Sci.*, **159**, 360.

WEINSTEIN, I. B., WIGLER, M. & PIETROPAOLO, C. (1977) The action of tumor-promoting agents in cell culture. In *Origins of Human Cancer.* Ed. Hiatt *et al.* New York: Cold Spring Harbor Press. p. 751.

WIGLER, M. & WEINSTEIN, I. B. (1976) Tumour promoter induces plasminogen activator. *Nature*, **259**, 232.